# Regulatory Barriers Blocking Standardization of Interoperability

**DOI:** 10.2196/mhealth.2654

**Published:** 2013-07-12

**Authors:** Daidi Zhong, Michael J Kirwan, Xiaolian Duan

**Affiliations:** ^1^Key Laboratory of Biorheological Science and Technology, Ministry of EducationBioengineering CollegeChongqing UniversityChongqingChina; ^2^DSheet LLCLeawood, KSUnited States; ^3^Chongqing Academy of Science & TechnologyChongqingChina

**Keywords:** medical device regulation, device interoperability, personal health device, standardization

## Abstract

Developing and implementing a set of personal health device interoperability standards is key to cultivating a healthy global industry ecosystem. The standardization organizations, including the Institute of Electrical and Electronics Engineers 11073 Personal Health Device Workgroup (IEEE 11073-PHD WG) and Continua Health Alliance, are striving for this purpose. However, factors like the medial device regulation, health policy, and market reality have placed non-technical barriers over the adoption of technical standards throughout the industry. These barriers have significantly impaired the motivations of consumer device vendors who desire to enter the personal health market and the overall success of personal health industry ecosystem. In this paper, we present the affect that these barriers have placed on the health ecosystem. This requires immediate action from policy makers and other stakeholders. The current regulatory policy needs to be updated to reflect the reality and demand of consumer health industry. Our hope is that this paper will draw wide consensus amongst its readers, policy makers, and other stakeholders.

## Introduction

The utilization of an interconnected mechanism to deliver personal health services has been widely recognized as an efficient way to improve people’s quality of life and reduce overall health care cost. By leveraging the latest consumer technologies from the information computer and telecommunication (ICT) domain, individuals are empowered to better manage their health and wellness, more effectively communicate with their service providers and ultimately, improve their personal health status and clinical outcomes while reducing their health care cost. To achieve this goal, we have to cultivate a healthy global industry ecosystem for the technology providers, its users, and operators, enabling them to create innovative personal health services. Due to the multidisciplinary nature of this ecosystem, developing a set of personal health device interoperability standards to ensure seamless cooperation between multiple stakeholders becomes a key prerequisite for this ecosystem.

International standard developing organizations (SDO) and industry alliances, such as Institute of Electrical and Electronics Engineers 11073 Personal Health Device Workgroup (IEEE 11073-PHD WG) [1], Bluetooth Special Interest Group (Bluetooth SIG) [2], and Continua Health Alliance (CHA) [3], are striving to develop, certify, and market the global interoperability standards for personal health devices and services. The outcome of this work has resulted in the publication of more than 20 international standards and more than 90 CHA-certified products available in the global market.

However, due to the lack of synchronization between personal health technology and the development of regulation and health policy, industrial experts in those SDOs have confronted non-technical barriers, which are difficult to achieve consensus within the SDOs. This has significantly delayed the massive market adoption of these health standards. Any inappropriate technical decision regarding these barriers may lead to insufficient-regulation or over-regulation towards personal health devices, both of which can impair the motivation of stakeholders and the health of the personal health industry ecosystem. This has become a global issue, recognized by several international industry alliances [3-6]. Policy makers, policy advisors, and vendors in many countries and regions are actively working on this policy gap, but with little success [7-9].

In this paper, we will discuss five topics. The first three topics mainly affect the market adoption of those interoperability standards; the remaining two topics have a direct impact on the content of those standards. Working together to achieve balanced solutions on these topics will significantly help SDOs to develop widely-adopted standards.

## Granularity of Regulation Policy

Currently, regulators mainly use "intended use" to determine whether a device is a medical device under regulation, based on the claims a device vendor makes about its product. This policy was introduced for traditional health care practices where the user of medical device is always the health care service provider. However, that is not well-suited to the current reality where individuals are empowered by personal health devices to become more active in managing their health. The same device has the potential to support both the health-oriented and medical-oriented use cases, depending on who uses it and how it is used. The boundary between them is blurred. From our perspective, the main differentiators between health- and medical-oriented use include the type of user, the environment, and purpose for using the devices. In fact, most of health devices currently available in the market fall into the definition of a medical device while others are in a gray area. For example, nearly all the publicly-marketed home-use blood pressure meters are registered medical device; however, the individuals who use them only use them to monitor their own health status and do not send their health data to their service provider. In this case, the device is a medical device but the operator of the device is not a clinician, the environment is not a clinical environment, and there may be hardware (eg, a mobile phone or tablet) and software (eg, a self-assisted health management app) associated with this blood pressure meter. Applying the same regulation policy that is usually applied to the traditional clinical-oriented medical devices over such type of health-oriented device and its associated software without properly differentiating its intended use may cause the situation where the increased cost cannot justify the benefit it brings. In order to fulfill the regulatory requirements, vendors need to dramatically increase their research and development (R&D) and productization cost to ensure their product is risk-free or risk-minimized as a traditional medical device. However, such type of health-oriented hardware and software are usually low acuity in terms of the risk it may cause to consumers. Many vendors believe such regulation is a kind of over-regulation, which may push up the product price, thus slowing down the market adoption. The ambiguity in the definition of a medical device and/or the words in the product’s claims themselves results in a lack of regulatory clarity and predictability for many personal health products. To solve this, granularity in the regulation policy is needed to distinguish the different types of uses.

As far as the device interoperability standard is concerned, the granularity about “intended use” alone is insufficient to eliminate ambiguity.

1. Devices for medical- and health-oriented use have quite dynamic requirements about the quality of service (QoS) of the underlying communication technology. “Realtime” data transmission is often required in clinical environment, and that is usually achieved via wired connection. In contrast, health devices often leverage communication technologies in consumer market, both wired and wireless. In the extreme case, some personal health applications (eg, a health watch connected to heart rate belt) may even work with connectionless broadcasting technology (eg, a sub-type of Bluetooth Low Energy (BLE) technology [2]), which basically provides no guarantee of data integrity, safety, or privacy. In this case, the benefit is the smaller implementation complexity, device size, power consumption, and R&D cost. Failing to acknowledge such differences may result in over-regulation.
2. Many countries and regions apply the same rule that an accessory of a medical device is subject to the same level of regulation as its “parent” device. The stack of interoperability standard is designed to carry medical data, thus is potentially regarded as an accessory of the medical device, and falls under the same regulation category of its associated device (eg, a glucose meter). However, the reality is the implementation of such standard (in hardware or software) only passively transmits health data, and such implementation is unlikely to cause similar risk as its associated device does. Applying the aforementioned rule over such accessories without enough differentiation will again introduce over-regulation, which may turn the chip vendors into medical device vendors.
3. Many consumer communication technologies are designed for general data transmission purposes. It is this dedicated application-level protocol (eg, IEEE 11073-PHD) that is running on top of the transport channel that turns the software stack into a health-oriented use. The simple wording like “data transfer” and “data conversion” in current regulation policy do not really cover such granularity, but this is crucial for consumer device vendors who implement platform for general purpose. These vendors may suddenly become vendors of medical device components and must go through, at least partially, the medical device regulation processes, which are generally considered as burdensome for them.

SDOs are expecting guidance with enough granularity from regulators, so as to tailor interoperability standards to better satisfy the technical, business, and regulatory requirements in each segment.

## Command and Control

One of these key barriers in question is the command and control (C&C) of personal health device. Many disease management services require, for example, that health device A sends a command to health device B in order to control B’s behavior. For example, a mobile phone sends command to Continuous Glucose Monitor to calibrate its glucose value, or a mobile phone sends commands to thermometer to change its measurement frequency from once per minute to once per five minutes. Depending on how it changes the behavior of the health device and how big the potential risk is, the devices (and associated software) supporting various C&C functionalities may be classified into different classification under medical device regulation. There is a general fear among consumer health device vendors with regard to the liability associated to such classifications, especially when something goes wrong during the data transmission and device operation.

So far, personal health device vendors only use proprietary methods to implement C&C. Vendors have differing opinions regarding whether they should or, if they agree, how to define C&C in a standard. Some vendors and users believe the current wireless communication technology cannot absolutely guarantee security. They believe that if C&C is standardized and implemented, the amount of adverse events will increase, and the regulation burden of these types of devices will be aggravated. There are however vendors and users who believe that standardizing C&C brings more benefit than risk, at least for a portion of personal health services. It’s unwise to block the beneficial portion just because the other portion may have risky components. In addition, the standardized technical solution generated based on collective wisdom of an entire industry is likely to be more secure and more sustainable than a proprietary solution. From a regulation authority’s perspective, regulating a proprietary technical solution has more opacity than a standardized one.

Currently, the consumer device vendors are eager to, but dare not to, use C&C functionality via standardized way due to the lack of clear regulatory guidance. In fact, both BLE and IEEE 11073 Point-of-Care standard series have already defined such functionalities, and similar work in IEEE 11073-PHD is also under going. But it is not likely that we will see this appear on the market in the near future. The large-scale field pilots probably will not begin in the market until the relevant regulation policy and public laws are defined and a complete legal framework dealing with these liability issues is established.

## The Selective Connection Between Peer Devices

The goal of building interoperable technical standards is to allow health devices to work together seamlessly in a plug-and-play manner. However, based on the market practice, some device vendors propose to standardize the functionality of selective connection complaining that when their devices get connected to a device manufactured by another vendor (especially some white-label device), the overall user experience may become very poor. One possible reason for that is the poor usability design from the questioning vendor. The way those vendors implement their products does follow the interoperable standards and guidelines, but unfortunately, in an inefficient or inappropriate way. One typical scenario is where the device repeatedly and unnecessarily sends connection requests to the peer device, which drains the peer device’s battery power quickly or disables the device’s ability to serve other devices normally.

In fact, it is technically feasible to define such functionality in current technical standard. But this is contradictory to the plug-and-play principle that these standards organizations are actively promoting. Consumers may easily get confused when two devices with same logo and cannot connect to each other. However, if the SDOs do not define this functionality, the user experience may become poor in certain scenarios, or this may further cause some technical risks. Lower layer communication standards often contain device filtering functionalities called the “white list” and “black list”. Such functionalities are usually requested by user, and are implemented by chip vendors. The health device vendors are not involved in this decision making process, thus their problems cannot be solved by the “white list” and “black list” functionality.

The user demand for customizing devices and services is another possible reason for selective connection. This is needed when particular health service providers wish to customize the health devices, so that these customized devices send health data only to certain designated service networks, rather than other service networks. Such customization can simplify the technical operation for aging people, and can ensure their personal health data goes directly to the designated service provider. However, if such functionality becomes a mandatory content of a standard, other users or service providers may be restricted from using the same health device. Given the competitive relationship between different vendors or service providers, the SDO is not likely to define functionality like “unconditional data redirection” in standards, even though it is technically feasible. What they can do is to standardize an attribute like “Preferred Destination” which may suggest a data flow direction. And this has to be an optional functionality that vendor may choose to not support.

## Identifiers of Device and Users

An end-to-end information system for personal health service often contains multiple health devices and different types of users. These devices and users are uniquely identified via identifiers (ID) within the system. These IDs identify the source of data; allowing the service provider to provide personalized service for clients via a set of devices, and the regulatory agency to backtrack all the devices and people involved in the information flow. This is quite useful when investigating medical adverse events. These IDs, typically device IDs (DID) and user IDs (UID), are important and necessary components of personal health device interoperability standards. Defining a proper way to use them in standards has significant impact to R&D cost, user experience, market entry, and regulation.

Nowadays, the DID defined in some mainstream communication standards (eg, the widely used IEEE EUI-64 identifier) actually identifies a particular piece of hardware providing the communication interface, thus indirectly identifies the entire device that embeds this communication hardware. There can be a device (eg, cpersonal computer) contains multiple communication interfaces, thus is associated to multiple DIDs.

In contrast, the DID usually identifies an entire device in medical device industry. It actually represents a combination of hardware, firmware, and application software. The Food and Drug Administration recently published the proposed rule for unique device identification (UDI) [10] , which aims to establish a UDI system for medical devices. But the UDI registration process seems to be inflexible and slow with regard to the evolving speed of consumer health products and services, because it requires each new version of device to have a new UDI. Software updates may happen frequently in consumer health devices and services. When a firmware update is urgently needed due to business or safety reason, vendors often cannot afford the waiting time to apply for a new UDI. This becomes more challenging when dealing with the international market where different types of DID exist. Therefore, the current IEEE 11073-PHD standards still rely on the EUI-64 being as its DID. SDO and vendors are expecting some harmonization of DID in the future. Ideally, that would be an “e-solution” which can balance above conflicts.

In personal health device use cases, scenario where multiple users share a single device (eg, weighing scale in home) often occurs. This requires the peer health device (eg, a mobile phone connected to that weighing scale) to process the mapping between DIDs and UIDs at application level. Due to this fact, that mobile phone and its mobile application involve deeply into the data flow, resulting in a higher level of medical device regulation. The regulation policy towards such ID-mapping case has not been explicitly clarified yet, which becomes one potential barrier for market entry. Both vendors and SDOs are expecting clear guidance about this issue from regulation authority.

## Duplicate Data

After the measurement channel has completed the measuring process, the captured health data is often stored in personal health device before uploading or clearing. For data transmission purpose, one measurement channel of a device can be associated to multiple logical transmission channels. The end result of that is the peer device may receive multiple copies of a single measurement data, either by duplicated transmission via the same channel, or by overlapped transmission via different channels. Some SDOs call this phenomenon as “duplicate data”.

According to current IEEE 11073-PHD standard, after the peer device (eg, a mobile phone) acknowledged the data reception, the personal health device (eg, a thermometer) can decide by itself whether to remove the successfully transmitted health data. This may happen when the device or device user delete data due to limited size of storage memory. While in BLE standard, health data is only transmitted once, thus it does not produce any duplicate data. Some vendors believe the peer device (which is receiving data) should be given the freedom to decide whether to merge the duplicate data. Another group of vendors believe the data merging process may aggravate the regulatory burden of the peer device, and may complicate and delay the data processing.

In fact, what is embedded in this data transmission process is an issue of transferring liability. So far, there is no regulatory policy which explicitly defines guidance for such duplicate data scenarios and the corresponding hand-over of responsibility of maintaining health data. The current consensus within PHD WG is: once the personal health device received the acknowledgment sent from the peer device, the peer device takes the responsibility to store and maintain the transmitted data. After that moment, any data lost or change in the uploading link has nothing to do with the personal health device that sent original data. Again, vendors and SDOs are expecting a clear guidance from regulation authority.

## Conclusion

Developing a set of personal health device interoperability standards is a mandatory prerequisite toward cultivating a healthy global industry ecosystem. However, the existing gaps between current regulations, health policies, and the resulting market reality hinder such progress. Policy makers must provide a predictable, risk-based regulatory framework that covers the issues arose from new market segment.

## Abbreviations

BLE: Bluetooth Low Energy
C&C: command and control
DID: device identifier
ICT: information computer telecommunication
ID: identifier
IEEE 11073-PHD WG: Institute of Electrical and Electronics Engineers 11073 Personal Health Device Workgroup
QoS: quality of services
R&D: research and development
SDO: standard developing organizations
UDI: unique device identification
UID: user identifier
